# Treatment outcome of new culture positive pulmonary tuberculosis in Norway

**DOI:** 10.1186/1471-2458-5-14

**Published:** 2005-02-07

**Authors:** Mohamed Guled Farah, Aage Tverdal, Tore W Steen, Einar Heldal, Arne B Brantsaeter, Gunnar Bjune

**Affiliations:** 1 Norwegian Institute of Public Health, Oslo, Norway; 2 Department of General Practice and Community Medicine, Faculty of Medicine, University of Oslo, Oslo, Norway; 3 Health and Welfare Agency, City of Oslo, Norway

## Abstract

**Background:**

The key elements in tuberculosis (TB) control are to cure the individual patient, interrupt transmission of TB to others and prevent the tubercle bacilli from becoming drug resistant. Incomplete treatment may result in excretion of bacteria that may also acquire drug resistance and cause increased morbidity and mortality. Treatment outcome results serves as a tool to control the quality of TB treatment provided by the health care system. The aims of this study were to evaluate the treatment outcome for new cases of culture positive pulmonary TB registered in Norway during the period 1996–2002 and to identify factors associated with non-successful treatment.

**Methods:**

This was a register-based cohort study. Treatment outcome was assessed according to sex, birthplace, age group, isoniazid (INH) susceptibility, mode of detection and treatment periods (1996–1997, 1998–1999 and 2000–2002). Logistic regression was also used to estimate the odds ratio for treatment success vs. non-success with 95% confidence interval (CI), taking the above variables into account.

**Results:**

Among the 655 patients included, the total treatment success rate was 83% (95% CI 80%–86%). The success rates for those born in Norway and abroad were 79% (95% CI 74%–84%) and 86% (95% CI 83%–89%) respectively. There was no difference in success rates by sex and treatment periods. Twenty-two patients (3%) defaulted treatment, 58 (9%) died and 26 (4%) transferred out. The default rate was higher among foreign-born and male patients, whereas almost all who died were born in Norway. The majority of the transferred out group left the country, but seven were expelled from the country. In the multivariate analysis, only high age and initial INH resistance remained as significant risk factors for non-successful treatment.

**Conclusion:**

Although the TB treatment success rate in Norway has increased compared to previous studies and although it has reached a reasonable target for treatment outcome in low-incidence countries, the total success rate for 1996–2002 was still slightly below the WHO target of success rate of 85%. Early diagnosis of TB in elderly patients to reduce the death rate, abstaining from expulsion of patients on treatment and further measures to prevent default could improve the success rate further.

## Background

The key elements in tuberculosis (TB) control are to detect the disease as early as possible and to ensure that those diagnosed complete their treatment and get cured. The World Health Organization (WHO) target for treatment success is 85 percent of all detected smear-positive cases [1]. Even where free medication is available, many patients are not successfully treated [2,3]. Main reasons for non-success are death (while on treatment or before start of treatment) and loss to follow-up. Incomplete treatment may result in prolonged excretion of bacteria that may also acquire drug resistance, cause transmission of disease and lead to increased morbidity and mortality [4].

Norway has about 4.5 million inhabitants, with foreign-born residents comprising 6.9% of the total population in 2002. The proportion of TB cases from foreign-born residents has increased from 19 % in 1986 to 76 % in 2002 [5]. However, the total number of cases has remained relatively stable. The reporting of treatment outcome for all TB cases has been obligatory since 1996. A study on treatment outcome for culture positive pulmonary TB in Norway in 1995 showed a high death rate and a high rate of loss to follow-up. Only 76% of patients who were included in that study completed treatment [6]. While the incidence rate of TB is low in Norway compared to most countries in the world, challenges still remain in achieving the WHO target for treatment success. However, there are some problems with the WHO definition of success rate and other measures have been proposed. A working group from the WHO, the International Union Against Tuberculosis and Lung Disease (IUATLD) and the Royal Netherlands Tuberculosis Association (KNCV) have defined a reasonable target for treatment outcome in low-incidence countries as to reduce the proportion of patients with a potentially bacteriologically unsuccessful outcome (failure, default, transfer) to less than 10% [7].

The aims of this study were to evaluate the treatment outcome for new culture positive pulmonary TB cases registered in Norway during the period 1996–2002, and to identify factors associated with non-successful treatment.

## Methods

### Setting and study population

This was a register-based cohort study. In Norway, there is compulsory nominative notification of all TB cases directly to the National TB Registry. Both suspected and confirmed cases have to be reported by clinicians. Laboratories of clinical microbiology are also required to report all isolates of *Mycobacteria*. A total of 25 microbiological laboratories isolate *M. tuberculosis *from patient samples. The laboratory at the Norwegian Institute of Public Health functions as a national reference laboratory for TB. The Pharmacy at the National Hospital is the only pharmacy distributing drugs for TB treatment, and TB prescriptions are compared with the National TB Registry in order to identify cases that may not have been notified. The notification is therefore considered to be quite complete [8]. There is also compulsory notification of all treatment outcomes to the Registry. Nine months after treatment start, the Register sends a special form to the clinician in charge to be filled out with details of the treatment outcome.

In this study, we included all new cases with culture positive pulmonary TB notified during 1996–2002. The sputum smear results were not recorded or reported for all cases, especially in the first years of the study period. We therefore based our analysis of treatment outcome on new cases with culture positive pulmonary TB.

The mode of detection was determined by information on the notification form. Data on susceptibility to TB drugs was obtained from the notification forms and laboratory reports. In addition to the information in the TB Registry, we used data from the Cause of Death Registry at Statistics Norway to determine the cause of death for those who died under treatment or before start of treatment.

Recommended regime for TB treatment in the study period consisted of isoniazid (INH), rifampicin and pyrazinamide in the intensive phase (two months). Ethambutol was added when resistance was suspected such as in foreign-born patients and in previously treated patients. The continuation phase consisted of four months with INH and rifampicin [9].

Directly Observed Therapy (DOT) was used before 2003 on an individual basis. However, we have no data to show how many patients in this study received DOT.

### Definition

The treatment outcome was divided into six categories according to WHO guidelines, with some modifications [10]. These categories were: *cured *(finished treatment with negative bacteriology result at the end of treatment), *completed treatment *(finished treatment, but without bacteriology result at the end of treatment), *failure *(remaining smear/ culture positive at five months despite correct intake of medication), *defaulted treatment *(patients who interrupted their treatment for two consecutive months or more after registration), *died *(patients who died due to TB or other cause before or during treatment), *transferred out *(patients whose treatment results are unknown due to emigration before or during treatment). Patients who changed treatment due to multi-drug resistant TB (MDR-TB), i.e. resistance to both INH and rifampicin, were also defined as failures [11].

Treatment success was defined as the sum of the cases that were cured and that completed treatment.

The proportion of patients with a potentially bacteriologically unsuccessful outcome (failure, default, transfer) was also calculated [7].

### Statistical analysis

We used the statistical package SPSS, version 11.0 for data analysis. To estimate the odds ratio for treatment outcome (success vs. non-success), logistic regression analysis was used. Confidence interval (CI) for the odds ratio has also been given. Variables such as sex, birthplace, age group, INH susceptibility, mode of detection and treatment periods (1996–1997, 1998–1999 and 2000–2002) were entered into both univariate and multivariate logistic regression model. P values of less than 0.05 were considered statistically significant.

## Results

Six hundred and fifty-five new culture positive pulmonary TB patients were included in the study (table 1). Of these, 397 patients (61%) were foreign-born. Three hundred and twenty three patients (49%) were cured and 221 patients (34%) completed treatment. This gives a treatment success rate of 83% (95% CI 80%–86%). The treatment success rates for women and men were 86% (95% CI 82%–90%) and 81% (95% CI 77%–85%) respectively. For those born in Norway and abroad, the rates were 79% (95% CI 74%–84%) and 86% (95% CI 83%–89%) respectively. The rates for the treatment periods 1996–1997, 1998–1999 and 2000–2002 were 83% (95% CI 77%–89%), 82% (95% CI 76%–88%) and 84% (95% CI 80%–88%) respectively.

**Table 1:** Number of new culture positive pulmonary tuberculosis patients by patient characteristics and treatment outcome, Norway, 1996–2002

	Cured	Completed treatment	Failure*	Defaulted treatment	Died	Transferred Out^†^	Total
Sex							
Women	144	90	2	7	17	11	271
Men	179	131	3	15	41	15	384
Birthplace							
Norway	119	84	1	5	49	0	258
Abroad	204	137	4	17	9	26	397
Age group (yrs)							
0–14	6	19	0	1	0	0	26
15–39	170	105	3	13	4	14	309
40–64	59	45	2	4	11	10	131
65+	88	52	0	4	43	2	189
INH-resistance^‡^							
No	295	210	0	15	53	22	595
Yes	21	10	5	7	2	4	49
Mode of detection^# ^(due to symptoms)							
Yes	221	154	3	16	47	12	453
No	100	67	2	6	11	13	199
Treatment periods							
1996–1997	67	48	0	3	19	2	139
1998–1999	83	58	1	7	20	3	172
2000–2002	173	115	4	12	19	21	344

*All patients are MDR-TB patients

^†^All patients have left the country

^‡^Information for INH susceptibility was available for 98.3% of the patients

^#^Information for mode of detection was available for 99.5% of the patients

In our study, the proportion of patients with a potentially bacteriologically unsuccessful outcome (failure, default, transfer) was 8%.

The average duration of residence in Norway for foreign-born patients at TB registration was 3.8 (range 1–31) years. There were no differences in the treatment success rates for those who had lived in Norway for less than three years and for those who had stayed longer than three years.

Among the 22 patients who defaulted treatment (3%) (table 1), four had MDR-TB and three additional patients had isolated INH resistant strains at the start of the treatment. The default rate was higher among foreign-born and male patients.

There were 26 patients (4%) who were transferred out (table 1). Of these, 21 were reported in 2000–2002. All of them have left the country. Twenty-two were on treatment when they left the country and four had not started treatment. Among the 26, seven were expelled from the country. Six of these were on treatment and one had not started treatment at the time of expulsion. For those who left the country while on treatment, the average duration of treatment was 78 days. Twelve of them had treatment only for two months or less. Of the 26 patients who were transferred out, three had isolated INH resistant strains and one had MDR-TB when they left the country.

Altogether 58 patients (9%) died, 19 (3%) before treatment start and 39 (6%) while on treatment. Of those who died before treatment start, four were diagnosed after death at autopsy. Eighty four percent of those who died were born in Norway. The median age for all who died was 80 years. For those who died before treatment start, the median age was 71 years. For those who died while on treatment, the median age was 81 years and the average duration of treatment was 67 days. Sixteen patients died within 14 days of treatment start. For 23 patients, TB was the primary cause of death. It was the only cause of death for nine of those 23 patients. For further 20 patients, TB was a contributing factor to their death. Fourteen patients died from other diseases than TB. The cause of death was unknown for one patient (table 2).

**Table 2:** Primary causes of death among new culture positive pulmonary tuberculosis patients, Norway, 1996–2002*

Tuberculosis mentioned on the death certificate	
Yes (N = 43)	No (N = 14))

Tuberculosis (N = 23)^†^	Heart failure (N = 1)
Acute myocardial infarction (N = 3)	Unspecified HIV disease (N = 1)
Heart failure (N = 2)	Unspecified non-Hodgkin's lymphoma (N = 1)
Unspecified cardiac arrest (N = 1)	Amyloidosis (N = 1)
Stroke, not specified as haemorrhage or infarction (N = 2)	Multiple myeloma (N = 1)
Chronic ishaemic heart disease (N = 2)	Mental and behavioural disorders due to use of opoids (N = 1)
Paroxysmal tachycardia (N = 1)	Mental and behavioural disorders due to multiple drug use and use of other psychoactive substances (N = 1)
Other chronic obstructive pulmonary disease (N = 1)	Malignant neoplasm of bronchus and lung (N = 5)
Unspecified respiratory failure (N = 1)	Liver cell carcinoma (N = 1)
Unspecified HIV disease (N = 1)	Other ill-defined and specified causes of mortality (N = 1)
Other acute viral hepatitis (N = 1)	
Unspecified sepsis (N = 1)	
Unspecified non-Hodgkin's lymphoma (N = 1)	
Spinal muscular atrophy and related syndromes (N = 1)	
Malignant neoplasm of breast (N = 1)	
Malignant neoplasm of bronchus and lung (N = 1)	

*The cause of death was unknown for one patient

^†^Including respiratory and miliary tuberculosis

There was no systematic registration of TB/ human immunodeficiency virus (HIV) co-infection at the TB Registry. For those who died while on treatment or before treatment started, HIV disease was registered as the cause of death for two patients (table 2).

Table 3 gives the odds ratio from logistic regression. The effect of birthplace in the treatment success changed from the univariate to the multivariate analysis. Patients who were born abroad had higher odds of success in the univariate analysis, but this changed to lower odds for success in the multivariate analysis. This is due to a strong confounding effect of age. The effect of age was distinct and graded, slightly stronger in the multivariate analysis. Both in the univariate and multivariate analysis, the odds for success were lower for those with INH resistant strains than for those with INH susceptible strains.

**Table 3:** Odds ratio (OR) for treatment success vs. non-success among new culture positive pulmonary tuberculosis patients notified in Norway, 1996–2002

	Treatment Success		Univariate		Multivariate*	
				
	Yes	No	OR	95% CI	OR	95% CI
Sex						
Women	234	37	ref.	ref.	ref.	ref.
Men	310	74	0.7	04–1.0	0.7	0.4–1.1
Birthplace						
Norway	203	55	ref.	ref.	ref.	ref.
Abroad	341	56	1.7	1.1–2.5	0.7	0.3–1.3
Age group (yrs)						
0–14	25	1	8.7	1.2–65.6	11.8	1.5–92.6
15–39	275	34	2.8	1.7–4.6	4.8	2.3–10.0
40–64	104	27	1.3	0.8–2.3	2.0	1.1–3.9
65+	140	49	ref.	ref.	ref.	ref.
INH-resistance						
No	505	90	ref.	ref.	ref.	ref.
Yes	31	18	0.3	0.2–0.6	0.2	0.1–0.4
Mode of detection (due to symptoms)						
Yes	375	78	ref.	ref.	ref.	ref.
No	167	32	1.1	0.7–1.7	1.1	0.7–1.7
Treatment periods						
1996–1997	115	24	0.9	0.6–1.6	1.2	0.7–2.2
1998–1999	141	31	0.9	0.5–1.4	0.9	0.5–1.5
2000–2002	288	56	ref.	ref.	ref.	ref.

*In the multivariate analysis, all variables in the univariate analysis were considered

Susceptibility testing for the main TB drugs was done in most of the patients. However, susceptibility testing for streptomycin was done for fewer patients than other main TB drugs. Resistance to INH and streptomycin was most common, and resistance was frequent among foreign-born patients. Of those with MDR-TB, two were born in Norway and eight abroad.

Of the 655 patients included in this study, 453 were detected through passive case finding (due to their symptoms). Another 101 patients were discovered through the immigration TB screening program. A total of 52 patients were detected through following ups of close contacts of identified infectious cases (22 cases) and of previous abnormal mass miniature radiology (MMR) (32 cases). The remainder was discovered through other screening programs. No information on mode of detection was available for three patients.

## Discussion

In our study, the total treatment success rate for new culture positive pulmonary TB for the period 1996–2002 was 83%. This is close to the WHO target of success rate of 85% of all smear positive cases. However, subgroups of patients contributing to low success rate warrant special attention such as those who defaulted treatment, those who were transferred out and those who died. The first two subgroups mainly comprise patients who were born abroad and the last subgroup mainly comprises patients who were born in Norway. Despite these problems, Norway has reached the reasonable target for treatment outcome in low-incidence countries [7].

Our study shows a default rate of 3%. Higher default rates have been described in other studies such as Vaud County, Switzerland (16%) [2], Hamburg, Germany (10%) [12], and Sweden (7%) [3]. Although the default rate in Norway is lower than in these countries, some of the patients who defaulted treatment, including patients with MDR-TB, have been the cause of small on-going outbreaks [13]. Default can constitute a major public health problem. Although incomplete treatment can prevent patients from dying from TB, the patients may remain infectious and even develop MDR-TB. It is therefore worrying that several patients in our study who defaulted treatment had isolated INH resistant strains or MDR-TB prior to treatment. Language problems, lack of understanding of the patients' cultural background, lack of communication between primary health care and hospitals, frequent change of address and stigma related to TB might be some of the reasons for defaulting. DOT was used on an individual basis during the study period, especially when an increased risk of non-adherence was suspected, but it became mandatory in Norway from 2003 according to the new TB regulations [14]. Adoption of this strategy will hopefully improve treatment adherence further.

In the transferred out group, the majority of patients left the country on their own initiative, but seven were expelled. Most of the patients who left the country were on treatment, but we do not have information about their treatment outcome. It is worrying that some of the patients who were expelled moved to countries with political unrest and poorly functioning TB programs. In this group three patients already had isolated INH resistant strains and one had MDR-TB. Expelling patients with TB before completion of treatment is unfortunate, unless it can be guaranteed that adequate treatment will be provided elsewhere. Efforts should be made to ensure the continuity of treatment for patients who move out of the country and, if possible, to allow them to start and complete their treatment, even if they have to leave the country later. The Netherlands have adopted a system where patients are not expelled from the country as long as they are on treatment. According to the new Norwegian manual for TB control and prevention [11], health personnel should encourage patients who are at risk to be expelled from the country to inform the Norwegian Directorate of Immigration through their legal representatives about their disease, and they may then be allowed to stay until treatment is completed. However, we believe there is a need for more awareness among health personnel, immigration authorities, the police and the legal representatives of the patients about this possibility.

The death rate in our study was 9%. Other studies from low TB incidence regions of the world showed death rates among TB patients of 24%, 14% and 6% in Baltimore City, USA [15], Vaud County, Switzerland [2] and Hamburg, Germany [12] respectively. Common for these studies and our study is that most patients who died were old, and many of them also had other illnesses. But if we only include only patients who started treatment, the actual death rate for our study was 6%. When considering the treatment outcome of TB, many studies including ours, include patients who never started treatment. This might seem contradictory, but it is an important issue that shows a deficiency in TB control. This is also in line with the recommendations of a working group of the WHO and the European Region of the IUATLD for uniform reporting by cohort analysis of treatment outcome in TB patients [16]. It has been suggested that acceptable treatment success rates need to be revised under such circumstances. It is difficult to know to what extent the death of the nine patients whose only cause of death was TB could have been prevented. Diagnosis of pulmonary TB can be especially difficult in older patients with co-existing illness. In one study, it was suggested that treatment of latent TB in such high risk elderly patients should be a high priority although advanced age is a relative contraindication [17]. Other studies recommend the start of anti-TB treatment on suspicion whilst awaiting results of diagnostic tests in elderly patients, provided there is no other obvious cause of their illness [18,19]. This makes sense as our study shows that 19 patients died before treatment start and four of them were diagnosed at autopsy. Two studies from Canada [20] and former Yugoslavia [21] have concluded that delay in diagnosis of TB was the main factor contributing to death from TB. Delay in diagnosis is outside the scope of this study. But autopsy rates in Norway are low [18] and therefore it is likely that there is under-diagnosis of deaths due to TB.

As indicated in the results section, TB/HIV co-infection is not a common cause of death for TB patients in Norway as most of the patients born in Norway were elderly persons with low risk of HIV and most foreign-born patients were from countries with low levels of HIV infection [22]. Therefore, we believe that it had a minor impact on the treatment outcome.

In the logistic regression model, increasing age and INH resistance were significant risk factors for non-successful treatment. We expected to find that age played a role, since old age in itself will contribute towards higher mortality, partly through co-existing illness. INH is a powerful bactericidal drug and resistance to the drug might reduce the effectiveness of standard short-course treatment [23]. Treatment was fairly standardized even in the previous TB manual from 1996: Four drugs to all foreign-born and to all previously treated TB patients and three drugs to patients born in Norway who were not likely to have been infected with drug resistant strains abroad. However, an analysis made by the National TB register in 1999 showed that only 75% of foreign-born patients received four drugs at the start of their treatment (Heldal E, personal communication, National TB Registry of Norway).

## Conclusion

Although the TB treatment success rate in Norway has increased compared to previous studies and although it has reached a reasonable target for treatment outcome in low-incidence countries, the total success rate for 1996–2002 was still slightly below the WHO target of success rate of 85%. Early diagnosis of TB in elderly patients to reduce the death rate, abstaining from expulsion of patients on treatment and further measures to prevent default could improve the success rate further.

## List of abbreviations

CI- Confidence interval

HIV- Human immunodeficiency virus

INH- Isoniazid

IUATLD- International Union Against Tuberculosis and Lung Disease

KCNV-Royal Netherlands Tuberculosis Association

MDR-TB- Multi-drug resistant tuberculosis, i.e. resistance to both isoniazid and rifampicin

TB- Tuberculosis

WHO- World Health Organization

## Competing interests

The author(s) declare that they have no competing interests.

## Authors' contributions

MGF participated in all phases of preparation of the manuscript (collection of data, analysis and interpretation of results and writing of the manuscript) and is corresponding author. AT, TWS, EH, ABB and GB have participated the interpretation of results and writing of the manuscript. All authors read and approved the final manuscript.

## Pre-publication history

The pre-publication history for this paper can be accessed here:



## References

[B1] World Health Organization WHO Tuberculosis Programme: Framework for Effective Tuberculosis Control. Geneva, Switzerland: WHO/ TB/ 94 179.

[B2] Zellweger JP, Coulon P (1998). Outcome of patients treated for tuberculosis in Vaud County, Switzerland. Int J Tuberc Lung Dis.

[B3] Romanus V, Julander I, Blom-Bulow B, Larsson LO, Normann B, Boman G (2000). Shortages in Swedish tuberculosis care. Good results only in 71 percent of cases after 12-month treatment as shown in a current study [in Swedish]. Läkartidningen.

[B4] Grzybowski S, Enarson DA (1978). The fate of cases of pulmonary tuberculosis under various treatment programmes. Bull Int Union Tuberc.

[B5] Winje BA, Heldal E (2003). Tuberculosis Disease in Norway 2002 [in Norwegian]. MSIS-rapport No 23.

[B6] Heldal E (1997). Results of tuberculosis treatment in Norway 1995 [in Norwegian]. Nor J Epidemiol.

[B7] Broekman JF, Migliori GB, Rieder HL, Lees J, Rutuu P, Loddenkemper R, Raviglione MC (2002). European framework for tuberculosis control and elimination in countries with a low incidence. Eur Respir J.

[B8] Heldal E (1995). Notification of tuberculosis in Norway [in Norwegian]. Nor J Epidemiol.

[B9] Bjartveit K (1996). The control of tuberculosis. A handbook for community health service [in Norwegian].

[B10] World Health Organization Global Tuberculosis Control. WHO Report 1999. Geneva, Switzerland, WHO/CDS/CPC/TB/99259.

[B11] The Norwegian Institute of Public Health (2002). Manual of tuberculosis control and prevention in Norway [in Norwegian].

[B12] Diel R, Nieman S (2003). Outcome of tuberculosis treatment in Hamburg: a survey, 1997–2001. Int J Tuberc Lung Dis.

[B13] Dahle UR, Sandven P, Heldal E, Caugant DA (2001). Molecular epidemiology of Mycobacterium tuberculosis in Norway. J Clin Microbiol.

[B14] The Royal Ministry of Health (2002). The Control of tuberculosis [in Norwegian].

[B15] Fielder JF, Chaulk CP, Dalvi M, Gachuhi R, Comstock GW, Sterling TR (2002). A high tuberculosis case-fatality rate in a setting of effective tuberculosis control: Implications for acceptable treatment success rates. Int J Tuberc Lung Dis.

[B16] Veen J, Raviglione M, Rieder HL, Migliori GB, Graf P, Grzemska M, Zalesky R (1998). Standardized tuberculosis treatment outcome monitoring in Europe. Eur Respir J.

[B17] American Thoracic Society and Center for Disease Control and Prevention (2000). Targeted tuberculin testing and treatment of latent tuberculosis infection. Am J Respir Crit Care Med.

[B18] Naalsund A, Heldal E, Johansen B, Kongerud J, Boe J (1994). Deaths from pulmonary tuberculosis in low-incidence country. Journal of Internal Medicine.

[B19] Rieder LH, Bloch AB, Snider DE (1991). Tuberculosis diagnosed at death in the United States. Chest.

[B20] Xie HJ, Enarson DA, Chao CW, Allen EA, Grzybowski S (1992). Deaths in tuberculosis patients in British Columbia, 1980–1984. Tuber Lung Dis.

[B21] Zafran N, Heldal E, Pavlovic S, Vukovic D, Boe J (1994). Why do our patients die of active tuberculosis in the era of effective therapy?. Tuberc Lung Dis.

[B22] Heldal E, Dahle UR, Sandven P, Caugant DA, Brattaas N, Waaler HT, Enarson DA, Tverdal A, Kongerud J (2003). Risk factors for recent transmission of *Mycobacterium tuberculosis*. Eur Respir J.

[B23] Nolan CM, Goldberg SV (2002). Treatment of Isoniazid-resistant tuberculosis with isoniazid, rifampicin, ethambutol and pyrazinamide for 6 months. Int J Tuberc Lung Dis.

